# Haematological Profile of Adults with Malaria Parasitaemia Visiting the Volta Regional Hospital, Ghana

**DOI:** 10.1155/2020/9369758

**Published:** 2020-02-11

**Authors:** Daniel Sakzabre, Emmanuel Akomanin Asiamah, Elliot Elikplim Akorsu, Albert Abaka-Yawson, Noble Dei Dika, David Annor Kwasie, Emmanuel Ativi, Confidence Tseyiboe, George Yiadom Osei

**Affiliations:** ^1^Department of Medical Laboratory Sciences, School of Allied Health Sciences, University of Health and Allied Sciences, Ho, Ghana; ^2^Laboratory Department, Volta Regional Hospital, Ho, Ghana

## Abstract

**Background:**

Malaria is known to cause severe health consequences due to its marked effects and alteration on the haematological parameters of infected individuals. This study evaluated the haematological profile of adult individuals infected with the malaria parasite.

**Methods:**

A retrospective study was conducted using archived data of malaria positive cases from January 2017 to March 15, 2019. Data retrieved included subjects' demographics, malaria parasite count, malaria parasite species, and full blood count parameters. A total of 236 malaria positive subjects were included in the study.

**Results:**

The study showed that more females were infected with the malaria parasite than males (69.07% and 30.93%, respectively). A total of 87.3% of the study population were infected with *Plasmodium falciparum* as compared to 12.7% infected with *Plasmodium malariae*. The commonest haematological abnormalities that were seen in this study were lymphopenia (56.78%), anaemia (55.51%), thrombocytopenia (47.46%), eosinopenia (45.76%), neutropenia (29.24%), monocytosis (21.19%), and leucocytosis (17.37%) in the infected subjects. The mean platelet count of *P. falciparum*-infected subjects was decreased as compared to the mean platelet count of *P. malariae*-infected subjects. There was a significant (*P* value <0.05) decrease in the number of platelet count with every unit increase in parasite density.

**Conclusion:**

Study participants infected with malaria demonstrated vital changes in haematological parameters with anaemia, thrombocytopenia, lymphopenia, monocytosis, and eosinopenia being the most important predictors of malaria infection especially with *P. falciparum* species.*P. falciparum*-infected subjects was decreased as compared to the mean platelet count of

## 1. Introduction

Malaria infection is a major public health problem in tropical areas. Global estimates suggest that the disease accounts for 300–500 million morbidity cases and contributes to approximately 3 million deaths annually [1]. Additionally, malaria parasitaemia is the leading cause of morbidity and mortality among children of the tropical and subtropical areas [2]. The burden of malaria remains a tropical health concern with Africa contributing to 91% of the cases recorded [2, 3]. The tropical regions are thus affected by malaria due to the suitable breeding conditions such as high humidity, high temperature, and significantly high amounts of rainfall as well as the numerous stagnant waters it is marked with, that supports the life cycle of the vectors (mosquitoes) which transmit the parasites [1]. Several species of the plasmodium parasite exist but only four are parasites of man which are *P. falciparum*, *P. ovale*, *P. vivax*, and *P. malariae*. *Plasmodium falciparum* is, however, the most predominant cause of malaria infection which results in over a million deaths in Africa alone [1, 4].

In Ghana, malaria is hyperendemic with *Plasmodium falciparum* accounting for 90–98% of the morbidity and deaths associated with the disease [5]. The plasmodium parasite is a blood parasite that causes haematological alterations. Some of the noted alterations are anaemia, thrombocytopenia, leucopenia, lymphocytosis, and infrequently, disseminated intravascular coagulation [3]. Malaria infection in humans is associated with a reduction in the haemoglobin level frequently leading to anaemia, of which the most severe cases are seen in *Plasmodium falciparum* [4]. Malaria infection also affects the haematopoietic physiology at any level and influences alterations in the haematological parameters resulting in numerous clinical presentations including anaemia [6]. These changes involve the major cell types such as red blood cells, leucocytes, and thrombocytes [7]. This study therefore evaluated the haematological profile of adult individuals infected with the malaria parasite.

## 2. Materials and Methods

### 2.1. Study Design/Eligibility Criteria

This was a hospital-based retrospective study, which sought to evaluate the haematological indices of all malaria parasitaemia cases at the Volta Regional Hospital Laboratory Department from January 2017 to March 15, 2019, where the data has been archived. The data retrieved included subject demographics, malaria parasite count, malaria parasite species, and full blood count parameters of adult (≥18 years) malaria parasitaemia cases. Individuals with malaria parasitaemia but were also infected with other conditions such as HIV, hepatitis B virus, or diabetes were excluded from the study.

## 3. Ethical Consideration

This study was approved by the Research Ethics Committee of the University of Health and Allied Sciences (UHAS-REC A.4 ׀242׀ 18-19). Permission was obtained from the Volta Regional Hospital management to use archived data from the laboratory's Health Administration and Management System. Subject's confidentiality was kept. The benefit of this study is the added literature in relation to effects of malaria infection on the haematological profile of infected persons.

## 4. Data Analysis

Data collected was analyzed using Microsoft Excel and Statistical Package for Social Science (SPSS) version 25.0. Categorical data were presented as frequency and percentages and noncategorical data were expressed as mean ± SD. This was then used to generate tables of both univariate and bivariate analysis. Kruskal–Wallis test and student's *t*-test were used to test for statistical difference between two means of nonparametric and parametric datasets, respectively, as in Tables 1 and 2. One-way ANOVA was used to determine statistical difference between the means of more than two independent variables as in Table 3. *P* value of ≤0.05 was considered statistically significant.

## 5. Results

### 5.1. Demographic Characteristics of the Study Participants

A total of 236 haematological profile reports for adult subjects aged 18 years and above who have been diagnosed with *Plasmodium* parasite infection were reviewed for this study. Of this, 73 were males with the remaining 163 being females, representing 30.93% and 69.07%, respectively. More than 75% of the subjects were within the age range of 18 to 44 years while less than 25% of them were above 44 years. A significant proportion (87.3%) of the subjects were diagnosed with *Plasmodium falciparum* infection whiles the remaining (12.7%) were diagnosed of *Plasmodium malariae* infection. Among the 73 males, 64 (87.67%) were infected with *Plasmodium falciparum* species and 9 (12.33%) were infected with *Plasmodium malariae* species. Similarly, the most predominant malaria species among the females was *Plasmodium falciparum* with a frequency of 142 representing 87.12%. The age group with the modal frequency of malaria subjects was 18–24 years with a total of 77 subjects representing 32.63% out of which 76 (98.70%) were infected with *P. falciparum* and 1 (1.30%) was infected with *P. malariae*, as shown in Table 4.

### 5.2. Haematological Profile of Study Participants

The review recorded that more than half of the subjects with *Plasmodium* parasitaemia were anaemic (55.51%) while 44.49% of them had normal haemoglobin concentrations. Also, more than 73% of the subjects recorded a normal range of WBC count with 17.77% having increased WBC count (leucocytosis). Of the total 236 malaria subjects, 112 presented with decreased platelet count (thrombocytopenia) and 118 presented with normal platelet count representing 47.46% and 50.00%, respectively. Neutrophilia was observed in 25 subjects whereas neutropenia was recorded in 69 subjects in proportions of 10.59% and 29.24%, respectively. Lymphopenia was markedly observed among the malaria diagnosed subjects (>56%). Furthermore, 68% of the subjects recorded normal values of monocytes and less than 10% had monocytopenia. Basophilia was also observed among 8 subjects whiles normal basophil count was recorded among 213 subjects representing 3.39% and 90.25%, respectively. Eosinopenia was recorded among 108 subjects and eosinophilia among 7 of them, representing 45.76% and 2.97%, respectively, as shown in Table 5.

### 5.3. Haematological Profile Based on Gender

The means (*x*) of haemoglobin, red blood cells, and haematocrit profiles were significantly higher (12.78 g/dl, 5.22×10^6^/*μ*L, and 36.86%), respectively, among the male subjects as compared to those of female counterparts (11.03 g/dl, 5.22×10^6^/*μ*L, and 32.75%), respectively. An approximate 4-unit difference of average haematocrit and 1 unit of both haemoglobin and red blood cells were observed within the two genders. Conversely, the means (*x*) of platelet and cell volume were recorded to be higher in females (169.8×10^3^/*μ*L and 86.57 fl), respectively, as compared to their male counterparts (159.7×10^3^/*μ*L and 79.61 fl) with more than 7-point difference. The red cell distribution width and mean cell haemoglobin concentration were also recorded to be 14.46%, 34.89 g/dl and 14.29%, 34.85 g/dl for male and female, respectively. Furthermore, slightly higher means of white blood cell and mean cell haemoglobin levels were recorded for the females as compared to the males (6.43×10^3^/*μ*L, 27.31 pg, and 7.00 ×10^3^/*μ*L, 29.92 pg, respectively) as shown in Table 1.

### 5.4. Haematological Profile Based on Type of Malaria Infection

Generally, this review observed that the unit difference among the various haematological parameters between the *P. falciparum*-infected group and *P. Malariae*-infected group were statistically insignificant except for platelets with *P* value of 0.0489, indicating that between the two species, the differences between the average concentration of platelets did not occur by chance. The rest of the haematological parameters had their *P* values to be greater than 0.05, indicating that the differences in their average concentrations between the two species were insignificant, hence occurred by chance (Table 2).

### 5.5. Haematological Profile Based on Parasite Density

Within the study, a very statistically significant decreasing number of platelets with every increased titre of parasite density from <1000 to >100000 was observed as compared to any of the other haematological parameters.

Neutrophil profile was also mildly decreased with an increasing titre of parasite load (<1000 to >100000). There was insignificant increase in white blood cells from <1000 to 10000 parasite loads with an abrupt rise at >100000 parasite density. An abrupt decrease in the means of haemoglobin, red blood cells, and red cell distribution width was observed at the highest titre of parasite load (>100000) with slight unit decrease in their mean values from <1000 to 100000 parasite densities. A slight increase in average monocyte counts was also recorded with increasing *Plasmodium* parasitaemia (Table 3).

## 6. Discussion

Malaria has shown to be a major public health concern in Africa which presents with numerous alterations in the subjects' haematological profile [2, 3].

This study is a two-year review of laboratory-diagnosed malaria cases from the Volta Regional Hospital comprising all laboratory-confirmed cases of malaria within this period.

This present study sought to investigate the effects of malaria on some haematological parameters among adults in Ho Municipality, Ghana.

The outcome of this study showed that females were more infected as compared to their male counterparts (69.07% and 30.93%, respectively), which is similar to the findings of a study by Mintaka & Opoku-Okrah [8], who also reported females to be more infected than males. The results obtained in this study is, however, contrary to studies conducted by Yadav et al. [9] which reported higher malaria parasitaemia in males (64.1%) than females (34.9%) and also by Adedapo et al. [10], which reported both adult males and females to be at the same risk of infection except for pregnant women who were stated to be at a higher risk. This disparity with other findings could be due to differences in geographical location, climate, population group, and size. The gender variations with regard to the outcome of the present study could be due to the health-seeking behaviour of males compared to their female counterpart whereby the former are more inclined to get their malaria medication over the counter as opposed to the latter who report to the hospital whenever they feel unwell. This study also recorded significant differences between the RBC, HGB, and HCT counts of the two genders where there were increases in these values for the male subjects as compared to the female subjects. This could be as a result of the difference in reference ranges between the genders.

In this study, more than 85% of the study population was infected with the *P. falciparum* species as compared to the other *Plasmodium* species, particularly *P. malariae*. This observation concurs with the outcome of a study by Kotepui et al. [11], which reported that infection with *P. falciparum* is more serious than any of the other malaria species. In this present study, 87.3% of the subjects were infected with the *P. falciparum* species, which corresponds with several other similar works by Nkumama et al. [12] and Squire et al. [5], which mentioned *P. falciparum* to be the commonest malaria species in sub-Saharan Africa, particularly Ghana. Haematological abnormalities are considered a hallmark of malaria and are reported to be most pronounced in *P. falciparum* infections as concluded in two different surveys by Dalrymple et al. [13] and Kotepui et al. [11]. There was, however, no recorded infection resulting from the other malaria species such as *P. vivax, P. ovale,* and *P. knowlesi* in this present study. This seeks to explain the observation by Snow et al. [14], who reported *P. falciparum* to be the predominant species in temperate regions.

The outcome of this study showed marked decrease in haemoglobin concentration to as low as <11 g/dl indicating anaemia in 55.51% of the reviewed cases. This finding is consistent with previous studies by Osaro et al. [2] and Maina et al. [6] which both attributed anaemia to malaria parasitaemia. Malaria-induced anaemia can result from several factors including the destruction of infected red blood cells which results in decreased RBC counts as well as the rapid removal of both parasitized and nonparasitized red blood cells. This is in consonance with a study by Bawah et al. [15] among children in Ho Municipality in which malaria-induced anaemia was also reported to be high.

This present study also demonstrated thrombocytopenia to be a major haematological change in malaria parasitaemia, where more than 47% of the study population showed decreased platelet count. This is in line with observations by Kotepui et al. [7], who in their work on the effects of malaria infection on haematological parameters also found a significant reduction in the platelet counts of more than half of their study population as well as findings by Srivastava et al. [3] which also stated increased thrombocytopenia among malaria subjects in Kenya. Srivastava et al. [3] also explained the pathophysiology of thrombocytopenia to involve the sequestration of platelets, splenic pooling of platelets, antibody- (IgG-) mediated platelet destruction, and adenosine diphosphate (ADP) release following haemolysis of parasitized RBCs, dysmegakaryopoiesis, platelet aggregation and activation, platelet invasion by parasites, platelet phagocytosis, platelet adhesion to erythrocytes, and oxidative stress. This study recorded a higher outcome (47.46%) of thrombocytopenia compared to thrombocytosis (2.54%). Hence, thrombocytopenia could be a reliable haematological parameter for diagnosing malaria.

White blood cells play an important role in the defense against malaria infection. This study showed a high percentage of leucocytosis (17.37%) and monocytosis (21.19%) as compared to leucopenia (8.47%) and monocytopenia (9.32%) which is in consonance with a study conducted by Bawah et al. [15], which also presented similar outcomes. Contrary to this study, Srivastava et al. [3] reported a higher percentage of leucopenia as compared to leucocytosis. Kotepui et al. [7] also recorded a decrease in the number of monocytes among their study group. This difference in outcomes could be as a result of other factors such as the severity of the disease, parasite density, co-infection, and immunity of the host as suggested by Ali et al. [16]. This present study also recorded a higher number of subjects with lymphopenia (56.75%) and eosinopenia (45.78%) as compared to lymphocytosis (8.9%) and eosinophilia (2.97%). Lymphopenia is usually profound but transient in acute malaria in nonimmune adults as stated in a work by Srivastava et al. [3]. According to Srivastava et al. [3], Fas-induced apoptosis is also a factor responsible for lymphocyte destruction leading to lymphopenia. No significant difference was recorded in the haematological profile of those infected with both the *P. falciparum and P. malariae* in this study except in their platelet number in which there was a significant variation in the two parasitized groups. The platelet count for *P. falciparum* and *P. malariae* was 163.60 × 10^3^/*μ*L and 214.20 × 10^3^/*μ*L, respectively. This present work noted a marked decrease in platelet count in those infected with the *P. falciparum* as compared to the *P. malariae*-infected subjects. This agrees with similar outcomes in studies conducted by Kotepui et al. [7] and Quintero et al. [17], which both indicated thrombocytopenia to be more common in *P. falciparum* infection than any of the other plasmodium species. Patterns between increasing parasite density and a significant decrease in the level of haematological parameters were observed in this study. Within this study, a significant decrease was observed in platelets and neutrophils with increasing parasite load (<1000 to >100000). Though insignificant, increase in total WBC count corresponded with increase in parasite density. Trends between increasing parasite density (<1000 to >100000) and decrease in RBC, HGB, and HCT counts and red cell distribution width were also observed in this present study. Alterations in these hematologic parameters with increased parasite density were also documented in a study by Kotepui et al. [11]. This study also recorded statistically insignificant pattern of increase in monocytes with increase in parasite density, which is contrary to the work conducted by Kotepui et al. [11].

The impact of malaria on haematological parameters which leads to anaemia could be due to the altered surface characteristics assumed by the RBCs which usually results in their destruction in the spleen, ultimately resulting in decreased haemoglobin, RBC, and HCT values.

## 7. Conclusion

Study participants infected with malaria demonstrated vital changes in most of the haematological parameters with anaemia, thrombocytopenia, lymphopenia, monocytosis, and eosinopenia being the most important predictors of malaria infection especially with *P. falciparum* species. When these haematological changes are used in combination with other clinical indicators and microscopy, the malaria diagnosis and treatment could be improved.

## Figures and Tables

**Table 1:** Haematological profile based on gender.

Parameter	Male	Female	*P* value
	Mean ± SD	Mean ± SD	
WBC (10^3^/*μ*L)	6.43 ± 3.04	7 ± 7.12	0.5137
RBC (10^6^/*μ*L)	5.22 ± 4.50	4.07 ± 0.715	0.0017
HGB (g/dL)	12.78 ± 2.56	11.03 ± 2.25	<0.001
HCT (%)	36.86 ± 7.27	32.72 ± 5.70	<0.001
MCV (fL)	79.61 ± 7.42	86.57 ± 10.26	0.4754
MCH (pg)	27.31 ± 5.53	29.92 ± 23.29	0.3456
MCHC (g/dL)	34.89 ± 2.81	34.85 ± 11.8	0.9759
PLT (10^3^/*μ*L)	159.7 ± 95.82	169.8 ± 92.29	0.4446
NEUT (10^3^/*μ*L)	3.75 ± 2.25	4.85 ± 7.93	0.2499
LYMPH (10^3^/*μ*L)	2.17 ± 3.44	2.29 ± 6.61	0.8832
MONO (10^3^/*μ*L)	0.59 ± 0.34	0.56 ± 0.35	0.4705
EO (10^3^/*μ*L)	0.10 ± 0.20	0.081 ± 0.15	0.4407
BASO (10^3^/*μ*L)	0.031 ± 0.035	0.047 ± 0.25	0.5707
RDW-CV (%)	14.46 ± 3.801	14.29 ± 2.57	0.6918

Data are presented as mean and standard deviation (SD) with *P* value to determine significance. *P* value <0.05 is significant, and *P* value >0.05 is insignificant.

**Table 2:** Haematological Profile based on type of malaria infection.

Parameters	*P. falciparum*	*P. malariae*	*P* value
WBC (10^3^/*μ*L)	6.73 ± 6.19	8.32 ± 5.47	0.3474
RBC (10^6^/*μ*L)	4.46 ± 2.7	4.04 ± 0.88	0.5613
HGB (g/dL)	11.59 ± 2.51	11.31 ± 1.98	0.691
HCT (%)	34.12 ± 6.45	32.28 ± 7.29	0.3056
MCV (fL)	80.27 ± 9.58	80.27 ± 7.74	0.9993
MCH (pg)	29.22 ± 20.19	27.32 ± 2.77	0.7258
MCHC (g/dL)	34.91 ± 10.2	34.06 ± 2.44	0.7547
PLT (10^3^/*μ*L)	163.60 ± 92.91	214.20 ± 89.67	0.0489
NEUT (10^3^/*μ*L)	4.56 ± 6.89	3.80 ± 3.02	0.6832
LYMPH (10^3^/*μ*L)	2.24 ± 5.96	2.38 ± 2.24	0.929
MONO (10^3^/*μ*L)	0.56 ± 0.34	0.63 ± 0.49	0.4583
EO (10^3^/*μ*L)	0.09 ± 0.17	0.09 ± 0.07	0.8829
BASO (10^3^/*μ*L)	0.04 ± 0.21	0.05 ± 0.06	0.9271
RDW-CV (%)	14.28 ± 3.02	15.42 ± 2.25	0.165

Data are presented as mean and standard deviation with *P* value to determine significance. *P* value <0.05 is significant, and *P* value >0.05 is insignificant.

**Table 3:** Haematological profile based on parasite density.

Parameter	<1000	1001–10000	10001–100000	>100000	*P* value
	Mean ± SD	Mean ± SD	Mean ± SD	Mean ± SD	
WBC (10^3^/*μ*L)	6.80 ± 5.19	6.83 ± 8.21	6.69 ± 3.342	8.12 ± 4.56	0.5178
RBC (10^6^/*μ*L)	4.33 ± 0.85	4.31 ± 4.02	4.20 ± 0.8465	3.61 ± 1.49	0.784
HGB (g/dL)	11.76 ± 2.93	11.55 ± 2.31	11.43 ± 2.147	10.13 ± 3.12	0.3775
HCT (%)	34.92 ± 6.34	33.69 ± 6.37	33.59 ± 6.008	29.87 ± 11.8	0.3773
MCV (fL)	80.64 ± 6.98	79.79 ± 10.26	80.17 ± 10.45	86.20 ± 13.17	0.6183
MCH (pg)	27.51 ± 3.3	31.00 ± 30.11	27.88 ± 7.475	31.90 ± 11.39	0.9795
MCHC (g/dL)	34.31 ± 1.7	34.23 ± 2.08	36.27 ± 18.64	36.45 ± 7.67	0.5495
PLT (10^3^/*μ*L)	205.40 ± 99.55	162.80 ± 87.53	137.10 ± 85.47	101.80 ± 52.17	<0.0001
NEUT (10^3^/*μ*L)	4.94 ± 8.58	4.44 ± 7.69	4.26 ± 2.053	3.59 ± 2.41	0.042
LYMPH (10^3^/*μ*L)	2.19 ± 3.94	2.44 ± 7.99	1.73 ± 1.858	1.15 ± 0.82	0.0623
MONO (10^3^/*μ*L)	0.54 ± 0.32	0.57 ± 0.34	0.59 ± 0.3975	0.62 ± 0.38	0.7888
EO (10^3^/*μ*L)	0.09 ± 0.11	0.10 ± 0.22	0.07 ± 0.129	0.07 ± 0.04	0.3813
BASO (10^3^/*μ*L)	0.02 ± 0.03	0.03 ± 0.04	0.09 ± 0.3863	0.03 ± 0.02	0.6139
RDW-CV (%)	14.63 ± 3.34	14.33 ± 2.71	14.49 ± 2.666	11.15 ± 5.66	0.8259

Data are presented as mean and standard deviation. *P* value <0.05 is significant.

**Table 4:** Demographic characteristics of the study participants.

Parameter	*Plasmodium falciparum*	*Plasmodium malariae*	Total
Gender			
Male	64 (87.67)	9 (12.33)	73 (30.93)
Female	142 (87.12)	21 (12.88)	163 (69.07)
Age			
18–24	76 (97.44)	2 (2.56)	78 (33.05)
25–34	66 (94.29)	4 (5.71)	70 (29.66)
35–44	29 (87.88)	4 (12.12)	33 (13.98)
45–54	15 (88.24)	2 (11.76)	17 (7.20)
55–64	17 (85.00)	3 (15.00)	20 (8.47)
≥65	16 (88.89)	2 (11.11)	18 (7.63)

Data are presented in figures and percentages.

**Table 5:** Haematological profile of study participants.

Parameter		Reference counts	Number (*N* = 236)	Percentage
Haemoglobin	Normal	≥12.5 g/dl	105	44.49
	Anaemia	<12.5 g/dl	131	55.51

Leucocytes	Normal	5.0–10.0 × 10^3^/*μ*l	174	73.73
	Leucocytosis	>10.0 × 10^3^/*μ*l	41	17.37
	Leukopenia	<5 × 10^3^/*μ*l	20	8.47

Platelets	Normal	150–400 × 10^3^/L	118	50
	Thrombocytosis	>450 × 10^3^/L	6	2.54
	Thrombocytopenia	<100 × 10^3^/L	112	47.46

Neutrophils	Normal	2.0–7.5 × 10^9^/*μ*l	139	58.9
	Neutrophilia	>7.5 × 10^9^/*μ*l	25	10.59
	Neutropaenia	<2.0 × 10^9^/*μ*l	69	29.24

Lymphocytes	Normal	1.0–4.0 × 10^9^/L	80	33.9
	Lymphocytosis	>4.0 × 10^9^/L	21	8.9
	Lymphopaenia	<1.0 × 10^9^/L	134	56.78

Monocytes	Normal	0.2–1.0 × 10^9^/L	162	68.64
	Monocytosis	>1.0 × 10^9^/L	50	21.19
	Monocytopaenia	<0.2 × 10^9^/L	22	9.32

Basophils	Normal	0.2–0.02 × 10^9^/L	213	90.25
	Basophilia	>0.2 × 10^9^/L	8	3.39
	Basopenia	<0.02 × 10^9^/L	15	6.36

Eosinophils	Normal	0.04–0.5 × 10^9^/L	121	51.27
	Eosinophilia	>0.5 × 10^9^/L	7	2.97
	Eosinopaenia	<0.04 × 10^9^/L	108	45.76

Data are presented in figures and percentages.

## Data Availability

Data used to support our findings are available with the corresponding author upon request.

## Additional Points

*Limitations and Recommendations*. Nutritional deficiencies, helminth infections, and haemoglobinopathies were not investigated in the present study. Thus, their contribution to the haematological changes observed in this study could not be ascertained.
